# ASCD: Automatic sensing and control device for crop irrigation scheduling

**DOI:** 10.1016/j.ohx.2024.e00523

**Published:** 2024-04-04

**Authors:** Noor Sabah Abbas, Muhammed S. Salim, Naseer Sabri

**Affiliations:** a Computer Engineering Department, Al-Nahrain University, Iraq; b Electronics & Communication Engineering Department, Al-Nahrain University, Iraq; c Technical Engineering College, Al-Farahidi University, Iraq

**Keywords:** Smart irrigation management, Scheduling irrigation, Soil moisture sensor, Atmega 2560

## Abstract

Traditional agricultural irrigation systems suffer from wasting a lot of water allocated for irrigation through evaporation and drainage. To address water waste and increase crop productivity, irrigation scheduling is used, as irrigation scheduling depends on four elements: soil moisture, soil characteristics, weather conditions, and plant type. This paper proposes a new design for monitoring agricultural conditions and controlling the amount of soil moisture by determining the required irrigation water ratios and the ideal time period for irrigating crops. An intelligent control and monitoring algorithm was created using experimental data for a wide range of plants (for soil moisture from 21 to 80 kPa), where plants were classified into three groups according to their common irrigation starting point. Based on the proposed plant classification, three models were provided, where irrigation ratios as the required parameter, soil moisture, plant type, and time interval as input parameters. This algorithm was used to program a custom-made automatic sensor and control device (ASCD). The ASCD is equipped with two input ports to communicate with two types of soil moisture sensors (a resistive sensor and a capacitive sensor) and with three output ports to drive three types of electronic irrigation valves. The ASCD can read from one sensor and drive one electronic valve at a time, the choice of which is made by the farmer. ASCD based the new algorithm showed high accuracy compared to a number of experimental results. The average absolute relative deviation (AAPD) of the new models and experimental data is 5.46 %. The application of the new algorithm shows a reduction in the amount of water used for crop irrigation during the day versus irrigation at night. On the other hand, ASCD has proven its success in sensing and controlling, and it works automatically and independently.

## Highlights

• An intelligent control and monitoring algorithm was created using experimental data for a wide range of plants (for soil moisture from 21 to 80 kPa).
• Based on the proposed plant classification, three models were provided, where irrigation ratios as the required parameter, soil moisture, plant type, and time interval as input parameters.
• This algorithm was used to program a custom-made automatic sensor and control device (ASCD).
• The ASCD is equipped with two input ports to communicate with two types of soil moisture sensors (a resistive sensor and a capacitive sensor) and with three output ports to drive three types of electronic irrigation valves.
• The average absolute relative deviation (AAPD) of the new models and experimental data is 5.46%. The application of the new algorithm shows a reduction in the amount of water used for crop irrigation during the day versus irrigation at night.
• On the other hand, ASCD has proven its success in sensing and controlling, and it works automatically and independently.

## Graphical abstract

**Figure:**
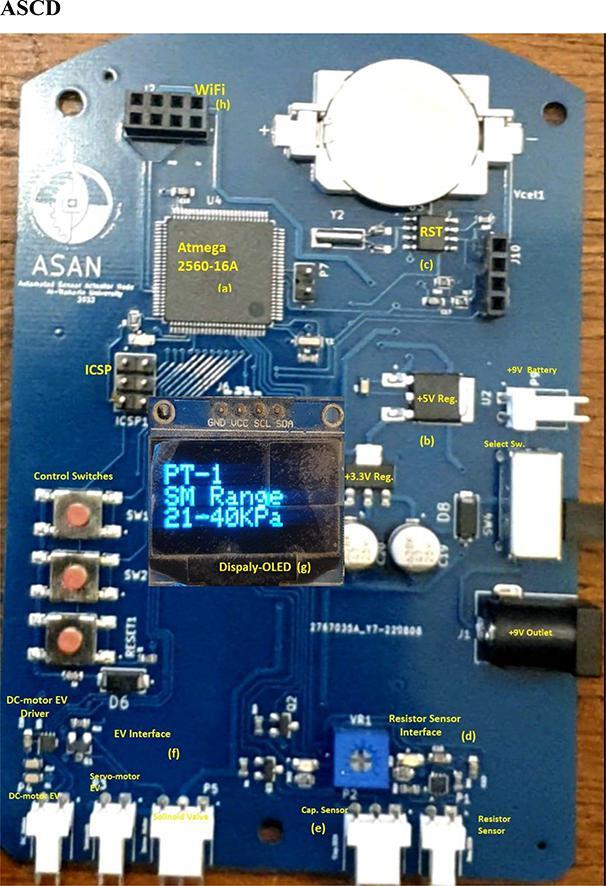

Specifications table

**Table:** 

Hardware name	*Automatic Sensing and Control Device (ASCD)*
Subject area	• Engineering and materials science • Environmental, planetary and agricultural sciences
Hardware type	• Measuring physical properties and in-lab sensors • Field measurements and sensors • Electrical engineering and computer science
Closest commercial analog	*No commercial analog is available.*
Open source license	CC BY 4.0
Cost of hardware	55.5461 USD
Source file repository	https://doi.org/10.17632/mgdnsbsxkt.1

## 1 Hardware in context

The effects of climate change and population growth have led to a decrease in the amount of water that can be used for agriculture. This is because filtration and evaporation cause irrigation water levels to drop. In order to produce the right crops, healthy soil and plants must be routinely watered at the right times and rates. Furthermore, proper water rates and timing are essential to achieving results properly. Often, manual irrigation systems are unable to manage irrigation water, especially in vast fields. Therefore, it was necessary to use smart irrigation systems because of their accuracy and high efficiency in managing irrigation water. Smart irrigation systems also accomplish the goal of using water in the most profitable way while keeping production levels at a sustainable level [1], [2], [3], [4], [5], [6], [7], [8], [9].

In this thesis, a proposed design for a sensor and control device that would use machine learning models trained on location-specific data to schedule crop irrigation is described. This would get rid of the need for unreliable models and expensive sensors and equipment. using machine learning models which are great at learning nonlinear relationships between parameters in a given dataset, to figure out how much and when to water a certain type of plant. Based on plant type and irrigation timing, the models will be used in the predictive modelling of a sensor and control device [10], [11], [12], [13], [14], [15], [16], [17].

In this work, an Automatic Sensing and Control Device (ASCD) based machine learning models is designed and assemble. It is an independent sensor, control, and actuator device, designed to be low-cost and have a small footprint. It can be compatible with a wide range of different plants, in addition to the possibility of modifying the old irrigation control units through its compatibility with two types of soil moisture sensors and three types of electronic valve drivers. This allows the module, like traditional watering devices, to control watering. The unit's operation, on the other hand, can be controlled based on the type of plant and precise run-time recommendations made by ASCD's actuation algorithm. The proposed working algorithm ensures that sufficient time is given for the water to soak into the soil before it is applied again.

The control is then given to the ASCD, which makes sure irrigation happens at the right time so that less water and electricity are wasted. In addition to the possibility of using ASCD as a sensor and actuating device within a wireless network. The device was placed in a weatherproof plastic box (12*12*8 mm) with IP64 to protect the ASCD mother board from harsh weather conditions in a farm, which, in previous cases, affected the circuitry and casing of the device. However, the sensors were able to perform under the harsh weather conditions as the temperature peaked at 52^◦^C in the summer.

## 2 Hardware description

**Fig. 1:**
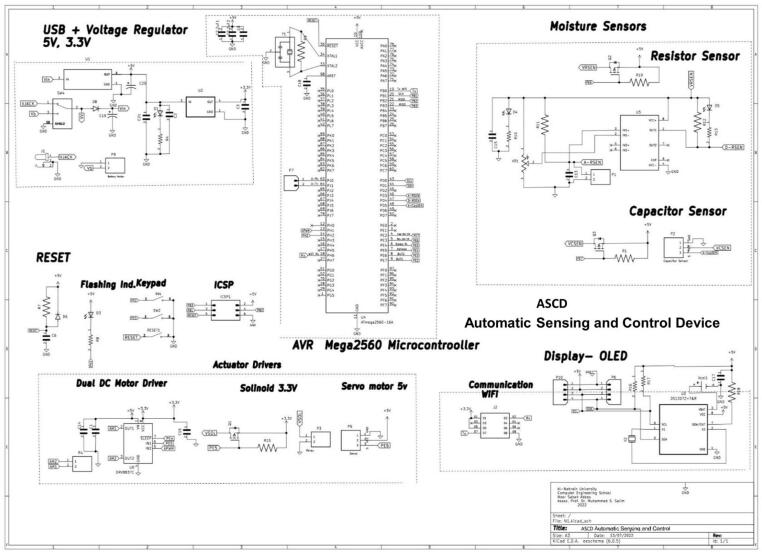
The schematic design of ASCD.

**Fig. 2:**
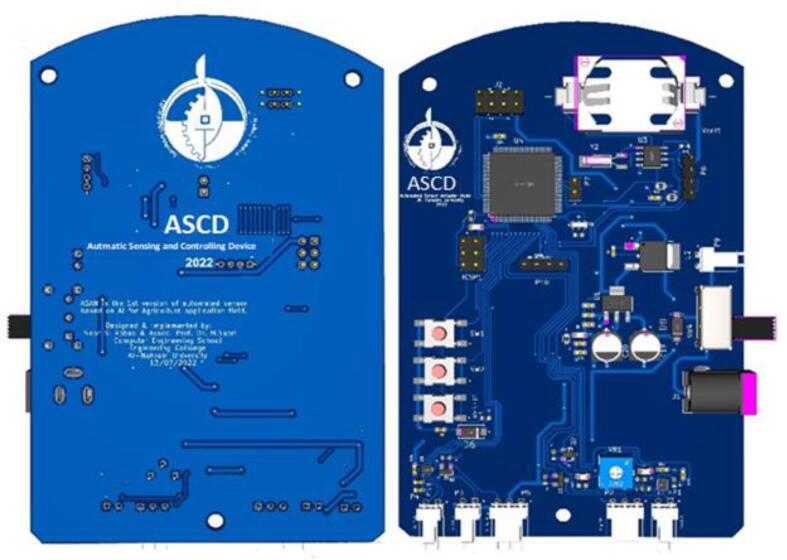
The ASCD prototype.

The schematic and prototype of the ASCD, depicted in Fig. 1 and Fig. 2 is equipped with a wireless sensor network protocol. ASCD is a low-cost and practical solution that can effectively replace older irrigation controllers. When the user selects the type of plant, the algorithm reads the amount of soil moisture, selects the appropriate predictive model, and then waters the plant with the amount of irrigation water required through electronic valve control [18], [19].

Optimizing irrigation saves water, money, and time while also protecting the environment. It is compatible with any drip or sprinkler irrigation pipe system, old or new. It has the following features:

a. Using moisture levels to control automatic irrigation.
b. Compatible with latching magnetic valves as well as electronic valve-based servo or DC motors.
c. This applies to any type of plant soil.
d. compatible with two types of soil moisture sensors (Resistive and capacitive sensor type).
e. Plant type can be selected via the organic light emitting diode (OLED) control screen.
f. LCD display that is simple to set up and use.
g. Design with a low power consumption.
h. Added a timer to allow the user to set wet/dry control levels as well as times when irrigation is not permitted.
i. is equipped with a wireless sensor network protocol

### 2.1 Power supply

**Fig. 3:**
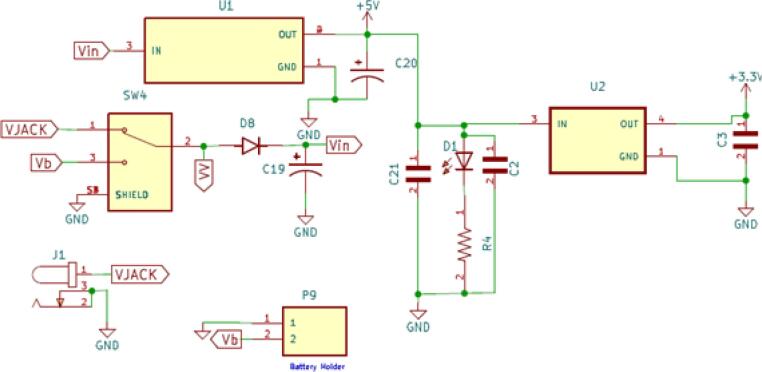
The schematic shows an ASCD-regulated voltage (5 V and 3.3 V) powered by either a 9–12 V DC power adapter or a 9 V rechargeable battery.

Fig. 3 shows the possibility of supplying power to the ASCD board either from the DC (9–12 V) power socket (J1) or a rechargeable battery (9) through the connector (P9). Using a 5 V regulator (U1) and a 3.3 V regulator (U2), the input voltage is decreased and regulated to 5 V and 3.3 V, which is needed for the ASCD and its accessories to work. The regulator output current must not exceed 1.0 A.

### 2.2 Processing unit

**Fig. 4:**
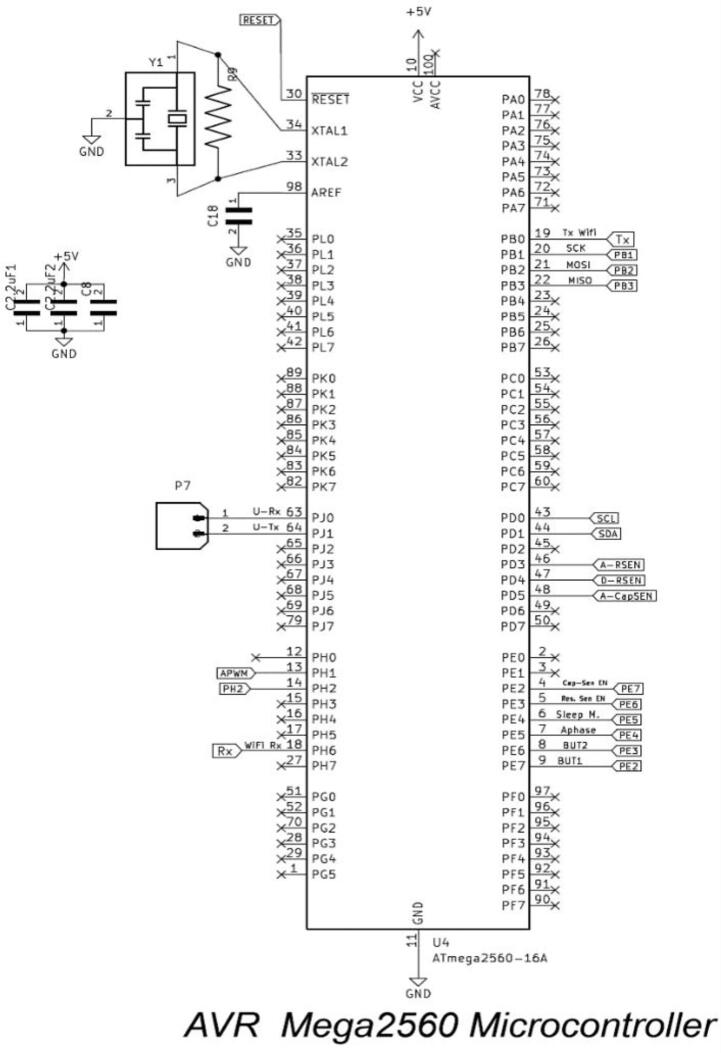
Pinout Configuration of ASCD's ATmega 2560 microcontroller.

A single task is done by an ATmega2560-16A (U4) microcontroller that is built into the ASCD control system, as shown in Fig. 4. ATmega16 is an 8-bit high-performance microcontroller in the Mega AVR family from Atmel. The Atmega16 microcontroller has 40 pins and uses a RISC (Reduced Instruction Set Computing) architecture that has been improved. It has 16 KB of programmable flash memory, 1 KB of static RAM, 512 bytes of EEPROM, and 10 bits of resolution (i.e. 1024 different values). It has a maximum frequency of 16 MHz. It does this by using its central processor to figure out what the data from the I/O peripherals means. In its data memory, the microcontroller stores the formulas for the predictive models, the algorithm for how it works, and any temporary information it gets. The processor can then use the instructions in its program memory and data memory to decode and use the incoming data. Then it uses its I/O peripherals to communicate and take the appropriate action. The ASCD’ microcontroller can be programmed using “in-circuit serial programming”. a serial protocol that allows the microcontroller to be programmed while it is still in the circuit.

The microcontroller receives soil moisture data through analogue or digital moisture sensors (a resistance sensor or a capacitive sensor). On the other hand, after processing, it sends a control signal to one of the three actuators types of electronics valve (solenoid, servo, or DC motor). in addition to interlocking it with the wireless transceiver unit.

### 2.3 ASCD sensing unit

The irrigation schedule is controlled by the sensor and actuating units built-in the ASCD. ASCD is built with two types of soil moisture sensors.

### 2.4 Resistive soil moisture sensor

**Fig. 5:**
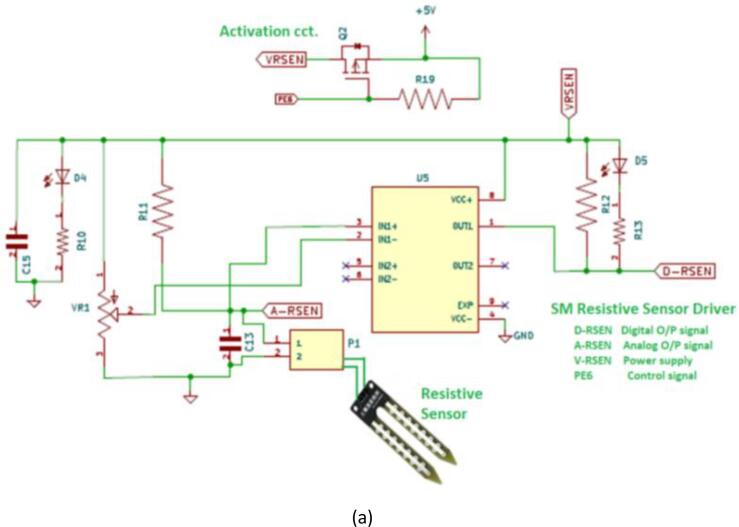

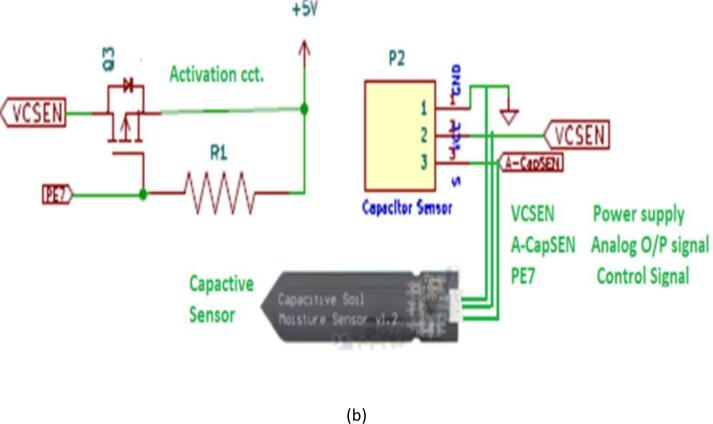
The ASCD offers two distinct ports for soil moisture measurement, allowing the farmer to choose between them: the first port is equipped with a built-in driver compatible with a resistive sensor (a), while the second port can be used to connect a capacitive sensor (b).

The first type measures soil moisture based on changes in the ground's electrical connection (soil resistance increases with dehydration). The electrical resistance between the sensor poles is measured. When the comparative exceeds an adjustable threshold, the digital or analogue output is activated. Table 1 shows the technical specification of the soil moisture resistive sensor type. A resistive sensor uses a continuous stream with two metal poles printed on a silicone plate. This causes electrical paint activity on one electric electrode and mineral erosion, which destroys the sensor for good over time. Fig. 5(a) shows the built-in ASCD resistive sensor driver circuit.

**Table 1:** The technical specification of Soil moisture sensors types.

SM Sensor Type	Operating Voltage (VCC)		O/P Signal		O/P voltage	Interface
	DC 3.3 V	DC 5 V	Digital	Analog		
Resistive	√	√	√	√	Depend on VCC	2-Pin
Capacitive	√	√		√	DC 0–3.3 V	3-Pin

### 2.5 Capacitive soil moisture sensor

Fig. 5(b) depicts the other type of soil moisture sensor; it’s an analogue capacitive soil moisture sensor that measures the volumetric amount of moisture in the soil. Unlike other moisture sensors, this capacitive one has the best build quality and is made of non-corrosive materials. A capacitive sensor produces analogue signals, and the metallic parts are not exposed to water, resulting in a longer time to run the sensor. This sensor module has an onboard voltage regulator that allows it to operate at voltages ranging from 3.3 to 5.5 V, making it compatible with all major microcontrollers.

### 2.6 ASCD actuating unit

**Fig. 6:**
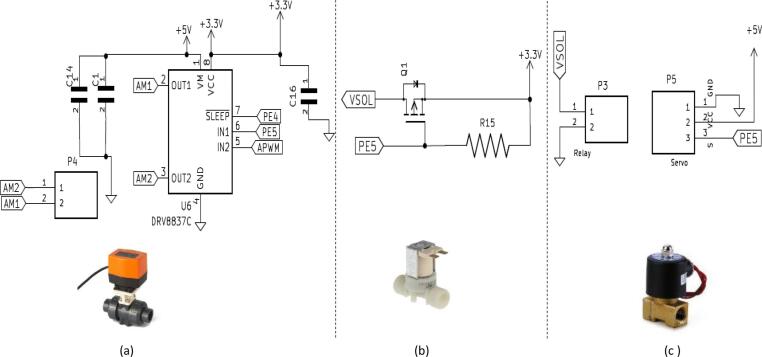
ASCD incorporates three varieties of actuator drivers:, (a) DC motor valves, (b) solenoid valves, and (c) servo motor valves, providing the farmer with enhanced options for selecting the desired actuator type.

ASCD is compatible with drip or sprinkler irrigation technology, so the output unit is designed to be compatible with three types of electronic valves, which are solenoids, DC motors, and servo motors Fig. 6. The proposed device provides farms with freedom of choice for one of the three electronic valves, depending on their abundance. The flow or stop of irrigation water is managed by an activation control signal issued by the microcontroller unit. where the microcontroller generates several distinct control signals for each input (sensing) and output (actuator) unit. The wake-up signal (PE4), direction signal (PE5), and speed signal (pulse width modulation (PWM)) are examples of control signals for opening or closing the DC motor electronic valve (U6).

### 2.7 Real time Clock, display and Wi-Fi units

The ASCD is equipped with an OLED screen that displays the device's operating system in text form. There are many types of OLED displays. They differ from each other in communication interface, sizes, and colors. The ASCD OLED display has a serial peripheral interface (SPI) communication interface, a 128x64 size, and a white color. In general, SPI is faster than Inter-Integrated Circuit (I^2^C) but requires more pins. Pins and communication speed are traded off. OLED displays are self-emitting light sources that don't need an external light source to display an image, unlike Thin Film Transistor (TFT) LCDs.

**Fig. 7:**
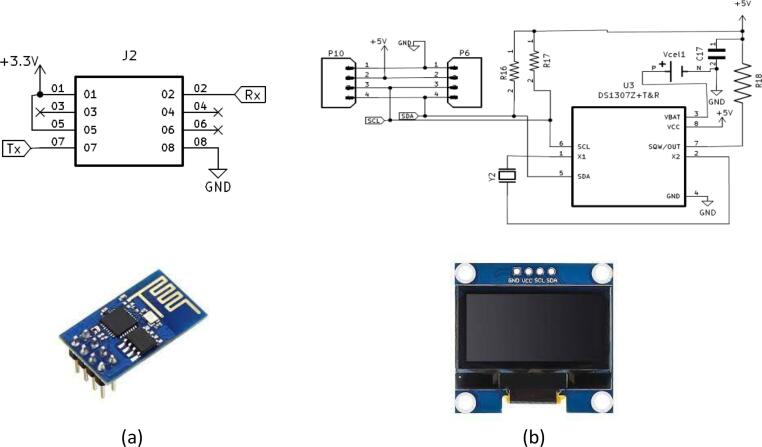
ASCD features an expansion ports for adding a wireless card (a), real-time clock and an OLED screen (b), enabling wireless connectivity and displaying findings.

Scheduling irrigation of agricultural crops is one of the objectives of this work, so a real-time clock (RTC) (built on ASCD) was used to determine the irrigation times during the day. A real-time clock (RTC) is an I2C device. RTC is a digital clock that keeps accurate time even when the power supply is turned off or the device is set to low-power mode. RTCs are made up of a controller, an oscillator, and a 16 MHz quartz crystal resonator embedded in the circuit as shown in Fig. 7. The ASCD can be connected to the Wi-Fi module for checking the operation of the device and the progress of the irrigation from a distance.

## 3 Design files summary

**Table:** 

Design file name	File type	Open source license	Location of the file
ASCD - Back capture.png	Image	CC BY 4.0	https://doi.org/10.17632/mgdnsbsxkt.1
ASCD - Front capture.png	Image	CC BY 4.0	https://doi.org/10.17632/mgdnsbsxkt.1
ASCD_Software.ino	.ino	CC BY 4.0	https://doi.org/10.17632/mgdnsbsxkt.1
List of Components.xlsx	Excel	CC BY 4.0	https://doi.org/10.17632/mgdnsbsxkt.1
N1.kicad_pcb	pcb	CC BY 4.0	https://doi.org/10.17632/mgdnsbsxkt.1
N1.kicad_sch	schematic	CC BY 4.0	https://doi.org/10.17632/mgdnsbsxkt.1
Figures 1–15	Figures	CC BY 4.0	https://doi.org/10.17632/9p6xwrjpb2.2
Table 3	Table	CC BY 4.0	https://doi.org/10.17632/9p6xwrjpb2.2
ASCD 1	MP4 File	CC BY 4.0	https://doi.org/10.17632/p5k7y76pmr.1

### 3.1 Bill of materials summary



## 4 Build instructions

A custom PCB was created using KiCad for the ASCD. For surface-mount device assemblies, use a hot air soldering iron to fill in the printed circuit board. Apply solder paste and flux to the bonding pad first, then place the components with tweezers, heat the components vertically at the position where the circuit board is weld components with a hot-air gun, preferably at a height of at least 3 cm to melt the solder paste and flux, and remove the hot-air gun immediately after the components are connected to the circuit board. After all of the components have been welded, clean the excess flux from the circuit board with plate washing water. Before soldering more significant SMD (Surface Mounted Devices) and inline type components, it is recommended to solder 0603 footprint capacitors, resistors, and various types of chips. Because larger chips or in-line components may cause the circuit board to be uneven, making other components difficult to solder. The front side of the board has various components of varying heights to be welded, and it is recommended that these components be welded in order of shortest to longest; the back side of the board does not contain electronic components.

## 5 Operation instructions

**Fig. 8:**
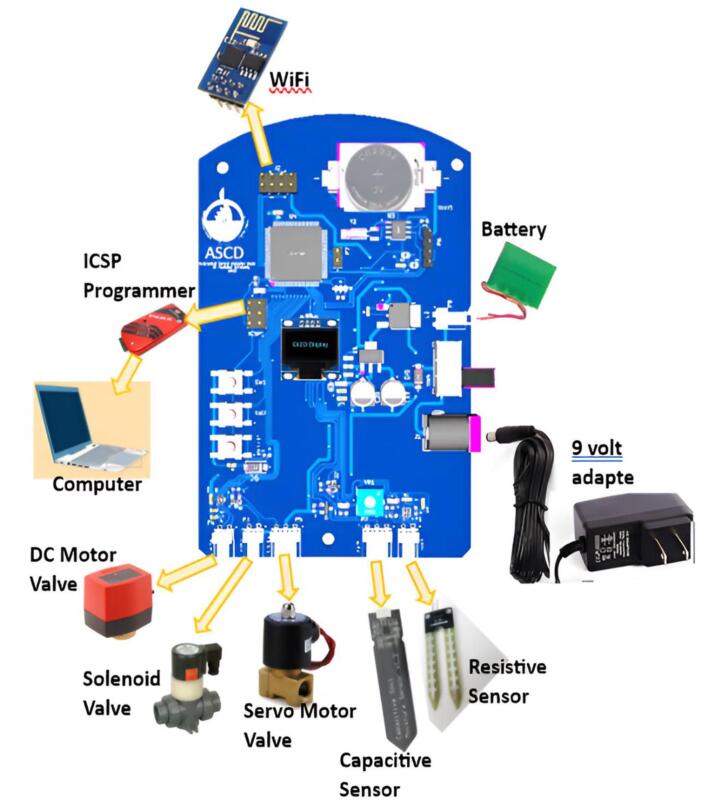
The external interface location of ASCD.

In general, the ASCD board can be used after connecting a stable external power source or by using 9 V rechargeable batteries, with one of the two sources selected by the switch, and Fig. 8 shows the connection position of the other interfaces. When the 9 V power supply is connected, one LED will light up, indicating that the 5 V and 3.3 V power supplies are working properly. If the LED1 does not light normally, inspect the power supply circuit. The resistive soil moisture sensor interface has two lines that read from right to left: analog signal and GND.

GND, 5 V, and Signal are the three capacitive sensor interface lines, from right to left. GND, Signal+, and 5 V are the three lines of the servo-motor valve interface, from right to left. 5 V and GND are the two lines of the solenoid valve interface, from right to left. From right to left, the two lines of the DC-motor valve interface are signal + and signal-.

### 5.1 Data preparation and operation algorithm

**Fig. 9:**
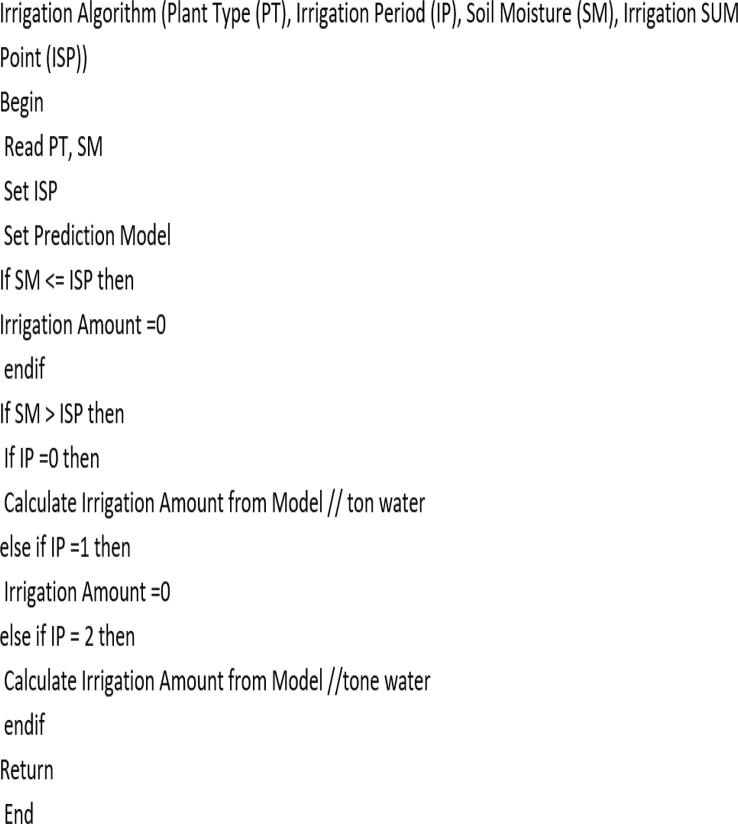
The proposed Irrigation Algorithm for calculation of output Irrigation Amounts.

There are two basic parameters of soil moisture measurement that describe the state of water in the soil. One is soil water content, and the other is soil water potential. Where Soil moisture measurement can be described through these two parameters. In this research the water potential (KPa) will be used as measurement unit of soil moisture. Plant irrigation was classified into three levels with specific ranges based on the starting point of irrigation, as shown in the Table 2. The running algorithm shown in Fig. 9 was generated using a set of 80 experimental data points for soil moisture (SM), irrigation time (IP), soil type (ST), and plant type (PT). Table 3 shows the amount of irrigation (IQ) calculated according to equation (1).

**Table 2:** Classification of plants-based irrigation start point.

Plant type examples	Irrigation start point	Model
Flowers, Strawberry, Apple	(21–40) KPa	M1
Blueberry, Lettice	(41–60) KPa	M2
Cranberry, Citrus, Tomato	(61–80) KPa	M3

**Table 3:** IQ calculation-based operation algorithm.

Inputs				Output	Set
SM	PT	ST	IP	IQ	
0–21	0	0	0	0	Set1

25	0	0	0	20	Set2
25	0	0	1	0	
25	0	0	2	75	

30	0	0	0	45	Set3
30	0	0	1	0	
30	0	0	2	100	

.					
.					
.					

100	0	0	0	395	Set16
100	0	0	1	0	
100	0	0	2	450	

The operating algorithm begins by requesting the type of plant, from which the algorithm identifies the required model (Table 2). The required model is determined, as there is no irrigation for less than the mentioned rates.

For example, if the plant type is a Rose plant (irrigation start point (ISP) is 21 KPa), the algorithm chooses the model (M1) whose humidity level of the rose plant falls within the range of this model (M1).

The watering time period (24 h) was divided into three periods in order to protect the plants from damage and save water.

During the first time period (IP = 0), which is the specified period from 6 AM to 5 PM, the plant was protected from damage during daytime hours by watered until the soil moisture reaches the required value of SM for the plant type (here for Roses, 21 kPa). While plants are not watered after 5:00 PM until 10:00 PM (IP = 1), the plant can protect itself until sunset by closing its stoma. During the third time period (IP = 2), which extends from 10:00 PM to 6:00 AM, where the water requirements of the soil are determined down to 10 kPa, which is accepted as the field capacity. As the parameters PT and ST were changed, they were set to a fixed value (PT assumed to be clay type). So, the developed working algorithm is suitable for a wide range of different plants.

(1) $$\mathrm{IQ}=\left\{\begin{array}{c} \left(SM-ISP\right)x5forIP=0\\ 0forIP=1\\ \left(SM-10\right)x5forIP=2 \end{array}\right)$$

### 5.2 ASCD firmware

**Fig. 10:**
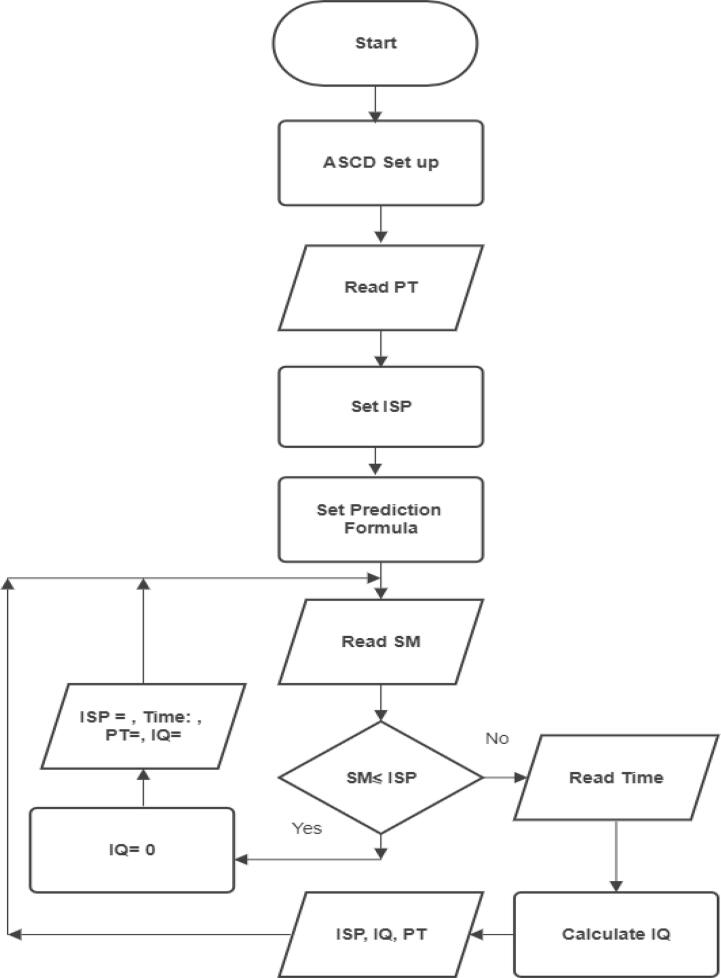
Program’s flowchart for scheduling irrigating process.

The ASCD’s control program is in charge of all device activities such as triggering sensors, running actuators, keeping time, recording, and updating, as well as communicating with the central server. The ASCD’s microcontroller is programmed to perform watering based on the type of plant the user chooses. The program then finds the plant's irrigation start point (ISP) and sets up a schedule for watering. Soil moisture levels are used to determine the amount and timing of irrigation water. A program subroutine will select the prediction formula for the previously defined plant type. Fig. 10 depicts the system's program flowchart. The OLED will display operation parameters such as irrigation water amount, currently soil moisture percentage value, irrigation start point and plant type.

### 5.3 Starting the device

1. Ensure power is supplied to ASCD. The first screen to display will be the logo, followed by welcome screen as seen in Fig. 11a.

**Fig. 11:**
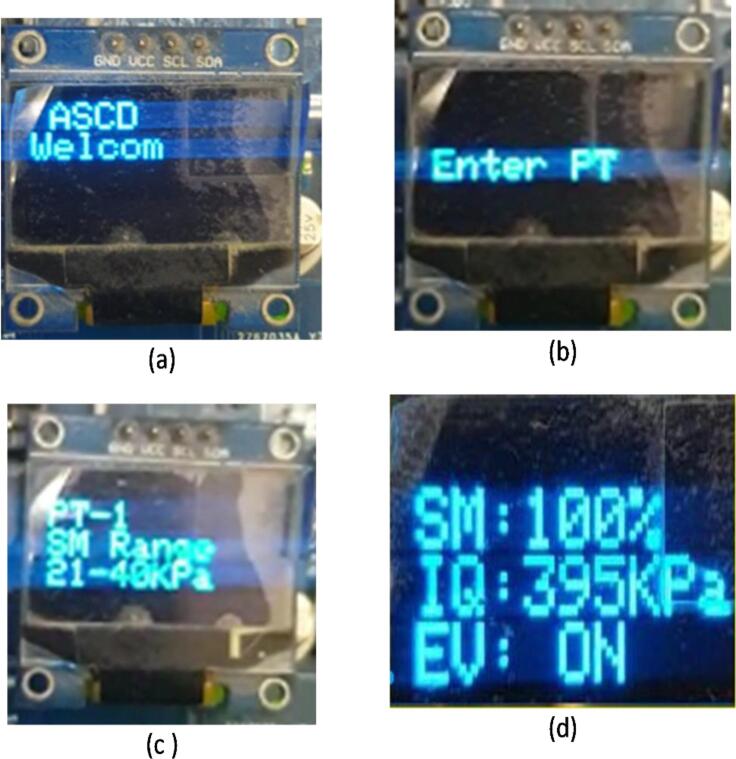
ASCD's OLED display shows operating steps.
2. Enter the plant type (see Table 2) (see Fig. 11b)
  i. Press the enter button (SW1) for < 0.5 s to select M1 model
  ii. Press the enter button (SW1) for < 0.9 s to select M2 model
  iii. Press the enter button (SW1) for > 0.9 s to select M3 model
3. Wait for 1 s, a new window that shows the information entered (see Fig. 11c)
  i. plant type, and
  ii. soil moisture level
4. The device starts working and a new window appears showing updated information for ASCD (see Fig. 11d)
  i. Measurement of real-time soil moisture
  ii. The amount of irrigation water required
  iii. Operating status of the electronic valve

## 6 Validation and characterization

This system is equipped with two kinds of moisture sensors (can use one type at a time), two kinds of power supplies, one solenoid valve, and two motorized electric valves (also, can use only one type of the three at a time). ASCD is intended for many plants such as organic vegetables, fruit trees and green grass, as it controls the amounts of irrigation water and the timing of irrigation according to the smart operating algorithm, so it can be said that the process of irrigating agricultural crops has been automated using such devices. The main functions of the device are as follows:

i. Select the type of plant and thus its IQ calculation model.
ii. Receiving moisture data through the soil moisture sensor.
iii. Using the IQ calculation model, calculate the amount of water needed for irrigation.
iv. Automatic control (ON/OFF) of the water pump (EV)
v. Display the IQ, EV state, Currently SM.

**Fig. 12:**
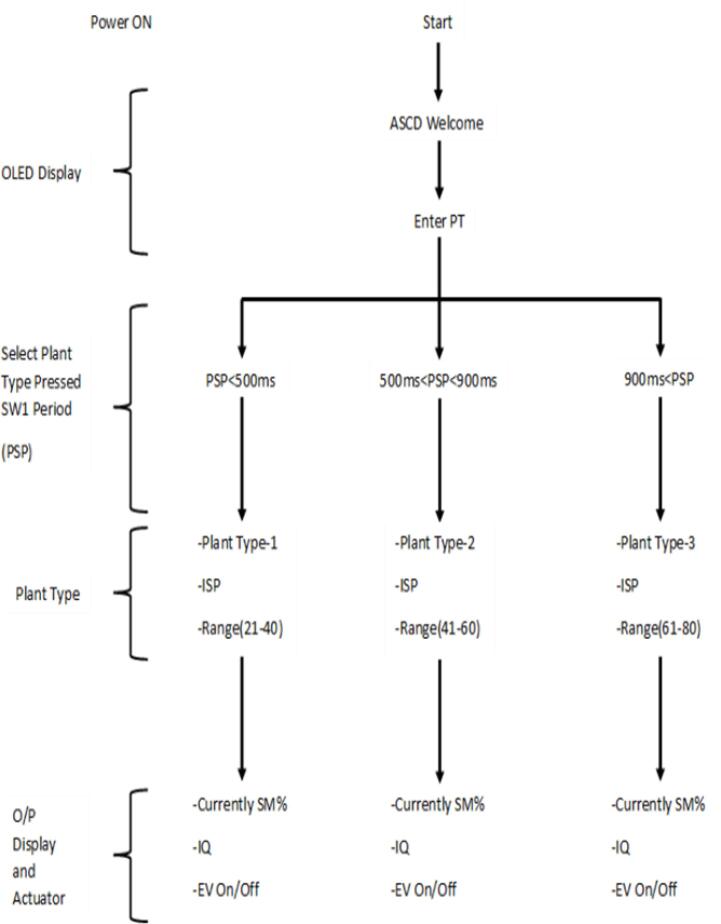
General flow operations of ASCD.

The general flow of operations through the device is described in Fig. 12 below.

The sensitivity of ASDC can be defined as a smallest absolute amount of change of soil moisture that can be detected by a measurement. ASCD analog input port by default can measure from ground to 5 V and ± 4 counts of noise, while the A/D converter provide 10 bits of resolution. So, the sensitivity will be (±5 counts × (2 ÷ 1024)) or about 10 mV. ASCD has a measurement time of < 10 ms, which makes it very suitable for monitoring rapid changes in soil moisture.

### 6.1 verification

**Fig. 13:**
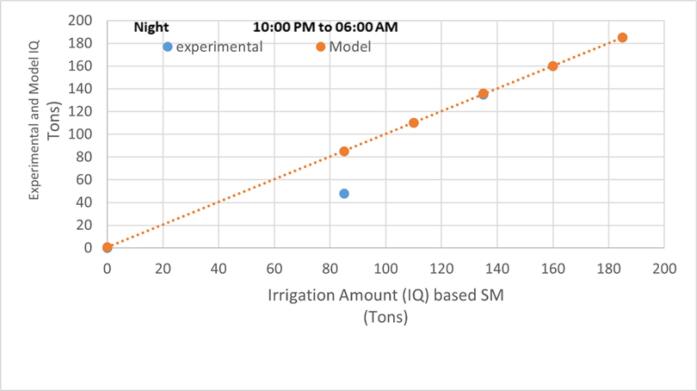
Comparison of experimental data and those calculated using the (M1) model for strawberry plants during IP = 2.

**Fig. 14:**
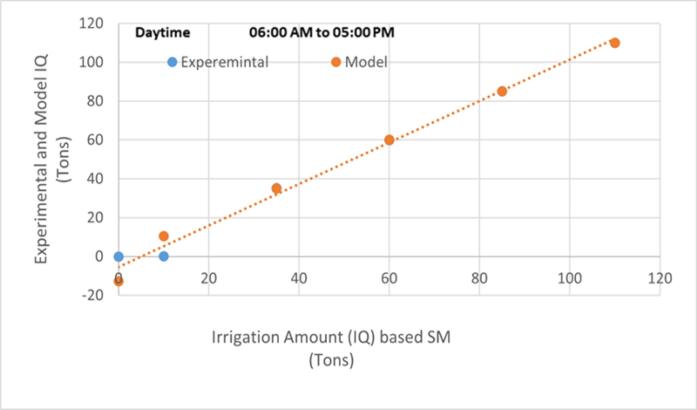
Comparison of experimental data and those calculated using the (M1) model for strawberry plants during IP = 0.

To verify the operation of ASCD and the accuracy of the proposed mathematical models (M1, M2, and M3), the device was installed in a strawberry field, where experimental data for day and night irrigation (10:00 PM- 6:00 AM and 6:00 AM-5:00 PM) were collected and compared with the results of the mathematical model (M1). The experiment demonstrated a high agreement between the field and theoretical results for the day and night irrigation periods, as shown in Fig. 13 and Fig. 14 as the average absolute coefficient of deviation (AAPD) for the model results (M1) and the experimental correlation is 5.46 %.

### 6.2 Second verification

The traditional farming methods currently used such as flood irrigation and the periodic water distribution system (warabandi) lead to excessive and inappropriate water use as well as low crop yields. The traditional method depends on the farmer's experience in determining the time needed to flood the field with water.

According to the farmer’s experience, it takes 20 h to flood the field with water, while 12 h needed for watering the field using ASCD, as the soil moisture was measured before watering and was found to be equal to 30 KPa.

Table 4, shows the total irrigation volume used in the strawberry field, as the field needed 200 tons of water over 20 h a day when using the traditional irrigation method. The amount of irrigation water using ASCD algorithm was 125 tons distributed by 25 tons during IP = 0 (morning) and 75 tons during IP = 2 (night), which represents 62.5 % of the irrigation water used in the traditional way. In other words, the irrigation scheduled by the ASCD algorithm saved 75 tons of water. This proves that irrigation scheduled using the ASCD algorithm, based on real-time farm parameters, optimizes the use of available resources and saves water without affecting crop yields. In addition, as a result of applying the proposed algorithm, there is an energy saving of 37.5 %.

**Table 4:** Comparison of the total irrigation volume and Energy required for strawberry field using traditional and ASCD.

Traditional Irrigation (Ton)	Energy Consumption (KW)	ASCD Irrigation (Ton)	Energy Consumption (KW)	Percentage of water saved	Percentage of energy saved
200	600	125	375	37.5 %	37.5 %

## 7 Conclusion

The aim of this paper was to reduce the waste of water used for the irrigation of agricultural crops. An automatic sensor and control system was designed implemented and put into place as a way to solve this problem. The ASCD design satisfies several engineering design constraints, such as economy or energy. ASCD distinguishes itself through its ease of installation and operation by the farmer, as well as its high accuracy in controlling the crop irrigation process. A New control algorithm was used to make a new model for calculating irrigation amounts based on the type of plant that would be watered on a new irrigation schedule. Three models were extracted to determine irrigation rates and timing for a wide range of plants (for soil moisture of 21–70 KPa), and these formulas were used to program a custom-made automatic watering device (ASCD). The ASCD can interlock with two types of sensors to determine how wet the soil is and work with three different types of electronic valves. in addition to the ability to work within a wireless sensor network. In addition, these models showed higher accuracy, where the mean absolute relative deviation (AAPD) of the new model and experimental data is 5.46 %. The application of the new algorithm shows a reduction in the amount of water used for crop irrigation during the day versus irrigation at night. On the other hand, ASCD has proven its success in sensing and controlling, and it works automatically independently or can be included to work within a wireless sensor network.

## CRediT authorship contribution statement

**Noor Sabah Abbas:** Writing – original draft, Visualization, Validation, Data curation. **Muhammed S. Salim:** Validation, Software, Methodology, Formal analysis, Data curation, Conceptualization. **Naseer Sabri:** Writing – review & editing, Supervision, Methodology, Investigation.

## Declaration of competing interest

The authors declare that they have no known competing financial interests or personal relationships that could have appeared to influence the work reported in this paper.
